# Supplementary material for: Causal Relationship between Aging and Anorexia Nervosa: A White-Matter-Microstructure-Mediated Mendelian Randomization Analysis
Source: Biomedicines. 2024 Aug 16;12(8):1874. doi: 10.3390/biomedicines12081874 (PMC11351342; doi:10.3390/biomedicines12081874)
Supplement: Supplementary file 1 [file biomedicines-12-01874-s001.zip › Supplementary Figure/Figure S4/Singe SNP plot of MR analysis for TL to ubm-b-1775.pdf]

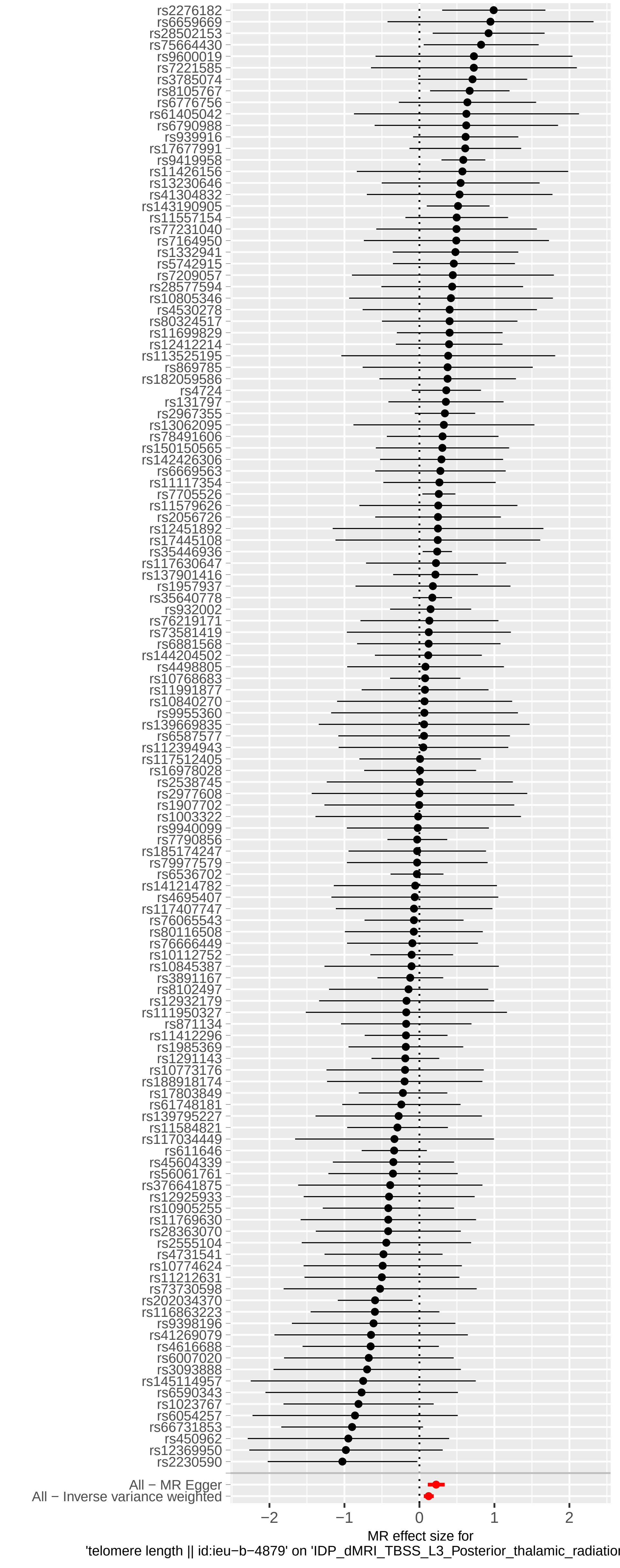
rs2276182
rs6659669
rs28502153
rs75664430
rs9600019
rs7221585
rs3785074
rs8105767
rs6776756
rs61405042
rs6790988
rs939916
rs17677991
rs9419958
rs11426156
rs13230646
rs41304832
rs143190905
rs11557154
rs77231040
rs7164950
rs1332941
rs5742915
rs7209057
rs28577594
rs10805346
rs4530278
rs80324517
rs11699829
rs12412214
rs113525195
rs869785
rs182059586
rs4724
rs131797
rs2967355
rs13062095
rs78491606
rs150150565
rs142426306
rs6669563
rs11117354
rs7705526
rs11579626
rs2056726
rs12451892
rs17445108
rs35446936
rs117630647
rs137901416
rs1957937
rs35640778
rs932002
rs76219171
rs73581419
rs6881568
rs144204502
rs4498805
rs10768683
rs11991877
rs10840270
rs9955360
rs139669835
rs6587577
rs112394943
rs117512405
rs16978028
rs2538745
rs2977608
rs1907702
rs1003322
rs9940099
rs7790856
rs185174247
rs79977579
rs6536702
rs141214782
rs4695407
rs117407747
rs76065543
rs80116508
rs76666449
rs10112752
rs10845387
rs3891167
rs8102497
rs12932179
rs111950327
rs871134
rs11412296
rs1985369
rs1291143
rs10773176
rs188918174
rs17803849
rs61748181
rs139795227
rs11584821
rs117034449
rs611646
rs45604339
rs56061761
rs376641875
rs12925933
rs10905255
rs11769630
rs28363070
rs2555104
rs4731541
rs10774624
rs11212631
rs73730598
rs202034370
rs116863223
rs9398196
rs41269079
rs4616688
rs6007020
rs3093888
rs145114957
rs6590343
rs1023767
rs6054257
rs66731853
rs450962
rs12369950
rs2230590
All − MR Egger
All − Inverse variance weighted
−2
−1
0
1
2
MR effect size for
'telomere length || id:ieu−b−4879' on 'IDP_dMRI_TBSS_L3_Posterior_thalamic_radiatio
